# Characterization of variations in IL23A and IL23R genes: possible roles in multiple sclerosis and other neuroinflammatory demyelinating diseases

**DOI:** 10.18632/aging.101058

**Published:** 2016-11-26

**Authors:** Fei-Feng Li, Xi-Dong Zhu, Peng Yan, Mei-Hua Jin, Hui Yue, Qiong Zhang, Jin Fu, Shu-Lin Liu

**Affiliations:** ^1^ Systemomics Center, College of Pharmacy, and Genomics Research Center (one of the State-Province Key Laboratory of Biopharmaceutical Engineering, China), Harbin Medical University, Harbin, China; ^2^ Translational Medicine Research and Cooperation Center of Northern China, Heilongjiang Academy of Medical Sciences, Harbin, China; ^3^ Department of Neurology, the Second Affiliated Hospital of Harbin Medical University, Harbin, China; ^4^ Department of Colorectal Surgery of the Second Affiliated Hospital, Harbin Medical University, Harbin, China; ^5^ Department of Antibiotics, Heilongjiang province food and drug inspection testing Institute, Harbin, China; ^6^ Department of Microbiology, Immunology and Infectious Diseases, University of Calgary, Calgary, Canada

**Keywords:** multiple sclerosis, IDD, interleukin-23A, gene expression level, SNP

## Abstract

Multiple sclerosis is among the most serious inflammatory demyelinating diseases (IDD). Interleukin-23A (IL23A) regulates and coordinates the activities of immune cells by interacting with its receptor IL23R and plays key roles in the pathogenesis of immune inflammatory diseases. IDD, deemed to be a kind of autoimmune diseases, may involve IL23A in the pathogenesis. The aim of this work was to validate the hypothesized involvement of IL-23A and its receptor in IDD. We sequenced the *IL-23A* and *IL-23R* genes for 206 Chinese Han IDD patients and evaluated SNPs within or near those genes. The serum levels of IL23A in IDD participants were analyzed using ELISA. The statistical analyses were conducted using Chi-Square Tests as implemented in SPSS (version 19.0). The Hardy-Weinberg equilibrium test of the population was carried out using online software OEGE. Three variants rs2066808, rs2371494, rs11575248 in *IL-23A* gene and one variant rs1884444 in *IL-23R* gene were demonstrated to be associated with the risk of MS or other IDD diseases, and the expression level of serum IL-23A in the MS patients was also altered. We conclude that variants in *IL-23A* and *IL-23R* genes were associated with the risk of MS or other IDD diseases.

## INTRODUCTION

Inflammatory demyelinating diseases (IDD) include a large group of nervous system disorders, such as multiple sclerosis (MS), neuromyelitis optica spectrum disorders (NMOSD) and acute disseminated encephalomyelitis (ADEM) [1], which have different clinical characteristics in terms of onset age, MRI and cerebrospinal fluid (CSF) features, clinical course and morbidity [1]. MS is considered the prototype [2] of IDD and affects over 2 million people worldwide [3]. The main manifestations of MS are transient and recurrent bouts of handicap[2], affecting mostly young adults with a strong sex bias of female to male about 4:1 [4], but the molecular mechanisms of IDD are essentially unclear.

Complex interactions between genetic and environmental factors play key roles in the inflammatory process of the diseases [2] or influence the susceptibility to these diseases [5]. Many lines of clinical and experimental evidence have shown the involvement of the immune system in IDD, especially MS [6]. So far, genetic linkage analyses and genome-wide association studies of MS have revealed that a large number of genes related to immune functions are associated with MS or other IDD [7]. Among the well investigated factors, interleukin-23A (IL-23A) is known to coordinate the activity of the immune cells and play a key role in the pathogenesis of immune inflammation diseases [8]. By supporting the Th17 cells, IL-23A is involved in chronic or autoimmune inflammations [9]. Additionally, IL-23A also has an important role in mediating some other autoimmune inflammation diseases, such as those in the brain and colon [10, 11]. When combined with the heterodimeric receptor, which consists of interleukin-23A receptor (IL-23R) and interleukin 12 receptor beta 1 (IL-12RB1), IL-23A performs its functions [12] and cells responding to the IL-23A signal are mainly determined by their expression of IL-23R [12]. Several variants in the *IL-23R* gene have been reported for their associations with human autoimmune disorders, such as psoriasis and inflammatory bowel disease [13, 14]. IDD, deemed a kind of autoimmune diseases, may involve IL23A in the pathogenesis.

In the present study, we analyzed the transcribed regions and splicing sites of the genes coding for IL-23A and its receptors IL-23R and IL-12RB1 and made comparisons between 206 Chinese Han IDD diseases (including 84 MS) and 300 controls. We also compared the serum levels of IL-23A in different genotype groups of the patients. Our results indicated that three variants rs2066808, rs2371494 and rs11575248 in the *IL-23A* gene and one variant rs1884444 in the *IL-23R* gene were associated with the risk of MS or other IDD diseases. Of considerable significance, the serum level of IL-23A in the MS patients was altered by the variants, strongly supporting the involvement of IL-23A in these diseases suggested by genomic analyses.

## RESULTS

### Clinical data

Two specialists in neurology at the Second Affiliated Hospital of Harbin Medical University, Harbin, China, conducted the diagnosis. There were no other systemic abnormalities in these IDD patients and they had no previous familial history of these diseases. The IDD patients (n = 206, male 60, female 146, the min and max ages were 15 and 73 respectively, and the average age was 41.45 years) included 84 MS, 14 myelitis, 8 NMO, 4 optic neyritis and 96 radiologically isolated syndrome (RIS) diseases that were not classified as MS, myelitis, NMO or optic neyritis. The recruited normal controls (n = 300, male 87, female 213, the min and max ages were 23 and 50 respectively, and the average age was 41.70 years) had no statistical differences in the gender composition or age with the IDD patients (Table 1).

**Table 1:** Clinical characteristics of study population

Parameter	IDD	Control	F	t	P	95%CI	
						Up	Low
**Sample (n)**	206	300	-	-	-	-	-
**Male/Female (n)**	60/146	87/213	-	-	0.975	-	-
**Age (years)**	41.45±13.30	41.70±7.06	115.938	-0.278	0.781	-2.04458	1.53778

Data are shown as mean±SD; between the two groups, there were no statistical differences of the age and gender composition

### SNP analyses

We sequenced the transcribed regions and splicing sites of the *IL-23A*, *IL-23R* and *IL-12RB1* genes to test the hypothesis that common genetic variants in those genes may confer the susceptibility to IDD diseases. We found variants within or near genes of *IL-23A* (rs2066808, rs2371494, rs11575248 and rs11171806), *IL-23R* (rs1884444, rs7530511, rs10889677 and rs76418789), and *IL-12RB1* (rs11575934 and rs401502). We compared genetic heterozygosity of the SNPs and found that the genetic heterozygosity of rs2066808, rs2371494, rs11575248, rs1884444, rs7530511, rs10889677, rs401502 and rs11575934 was very high (Fig. S1), whereas that of rs11171806 and rs76418789 was very low.

### Polymorphism-disease association analyses

In order to test any possible associations between *IL-23A*, *IL-23R* or *IL-12RB1* and IDD diseases, we conducted analyses on the SNPs and found that the variants rs2066808, rs2371494, rs11575248 in the *IL-23A* gene and rs1884444 in the *IL-23R* gene were associated with the risk of IDD diseases in the Chinese Han population (Tables 2, 3); however, the variants rs7530511, rs10889677 in the IL-23R gene and rs401502, rs11575934 in the IL-12RB1 gene were not. Of special significance, the variants rs2066808, rs2371494, rs11575248 and rs1884444 were closely associated with the risk of MS but not RIS (Tables 2, 3).

**Table 2:** The genotype and allele frequency of rs2066808, rs2371494, rs11575248 and rs1884444 variants in 206 Chinese Han Neuroinflammatory demyelinating diseases of the central nervous system patients and 300 non- IDD controls

Genes	Variants	Group		Genotype frequency (%)			Allele frequency (%)	
***IL23A***	Rs2066808	Genotype		T/T	T/C	C/C	T	C
		IDD	206	183(88.8)	23(11.2)	0(0.0)	389(94.4)	23(5.6)
		MS	84	72(85.7)	12(14.3)	0(0.0)	156(92.9)	12(7.1)
		RIS	96	87(90.6)	9(9.4)	0(0.0)	183(95.3)	9(4.7)
		Controls	300	287(95.7)	11(3.7)	2(0.7)	585(97.5)	15(2.5)
	Rs2371494	Genotype		C/C	C/A	A/A	C	A
		IDD	206	180(87.4)	26(12.6)	0(0.0)	386(93.7)	26(6.3)
		MS	84	72(85.7)	12(14.3)	0(0.0)	156(92.9)	12(7.1)
		RIS	96	85(88.5)	11(11.5)	0(0.0)	181(94.3)	11(5.7)
		Controls	300	283(94.3)	16(5.3)	1(0.3)	582(97.0)	18(3.0)
	Rs11575248	Genotype		C/C	C/A	A/A	C	A
		IDD	206	182(88.3)	24(11.7)	0(0.0)	388(94.2)	24(5.8)
		MS	84	71(84.5)	13(15.5)	0(0.0)	155(92.3)	13(7.7)
		RIS	96	86(89.6)	10(10.4)	0(0.0)	182(94.8)	10(5.2)
		Controls	300	283(94.3)	17(5.7)	0(0.0)	583(97.2)	17(2.8)
***IL23R***	Rs1884444	Genotype		T/T	T/G	G/G	T	G
		IDD	206	76(11.0)	108(74.0)	22(15.1)	260(63.1)	152(36.9)
		MS	84	35(41.7)	42(50.0)	7(8.3)	112(66.7)	56(33.3)
		RIS	96	33(34.4)	52(54.2)	11(11.5)	118(61.5)	74(38.5)
		Controls	300	101(33.7)	133(44.3)	66(22.0)	335(55.8)	265(44.2)

IDD: inflammatory demyelinating diseases; MS: multiple sclerosis; RIS: radiologically isolated syndrome.

**Table 3:** Associations of rs2066808, rs2371494, rs11575248 and rs1884444 variants within *IL23A* or *IL23R* with risk of IDD in Chinese populations

Titles				Pearson Chi-square				Pearson's R			
Genes	Genotyped SNP	Diseases	Statistical Types	Value	Min count^a^	df	Asymp. Sig. (2-sided)	Value	Asymp. Std. error^b^	Approx. T^c^	Approx. Sig
***IL23A***	Rs2066808	IDD	Genotype	12.207	0.81	2	0.002	-0.109	0.047	-2.459	0.014^d^
			Allele	6.422	15.47	1	0.011	-0.080	0.032	-2.540	0.011^d^
		MS	Genotype	13.610	0.44	2	0.001	-0.139	0.062	-2.752	0.006^d^
			Allele	8.341	5.91	1	0.004	-0.104	0.044	-2.900	0.004^d^
		RIS	Genotype	5.528	0.48	2	0.063	-0.072	0.056	-1.441	0.150^d^
			Allele	2.369	5.82	1	0.124	-0.055	0.040	-1.539	0.124^d^
	Rs2371494	IDD	Genotype	9.148	0.41	2	0.010	-0.113	0.046	-2.546	0.011^d^
			Allele	6.438	17.91	1	0.011	-0.080	0.032	-2.543	0.011^d^
		MS	Genotype	8.020	0.22	2	0.018	-0.123	0.060	-2.428	0.016^d^
			Allele	6.001	6.56	1	0.014	-0.088	0.043	-2.456	0.014^d^
		RIS	Genotype	4.584	0.24	2	0.101	-0.087	0.056	-1.726	0.085^d^
			Allele	3.071	7.03	1	0.080	-0.062	0.040	-1.754	0.080^d^
	Rs11575248	IDD	Genotype	5.873	16.69	1	0.015	-0.108	0.045	-2.433	0.015^d^
			Allele	5.625	16.69	1	0.018	-0.075	0.032	-2.376	0.018^d^
		MS	Genotype	8.768	6.56	1	0.003	-0.151	0.061	-2.988	0.003^d^
			Allele	8.412	6.56	1	0.004	-0.105	0.044	-2.912	0.004^d^
		RIS	Genotype	2.583	6.55	1	0.108	-0.081	0.056	-1.608	0.109^d^
			Allele	2.492	6.55	1	0.114	-0.056	0.040	-1.579	0.115^d^
***IL23R***	Rs1884444	IDD	Genotype	11.043	35.83	2	0.004	-0.102	0.043	-2.298	0.022^d^
			Allele	5.334	169.77	1	0.021	0.073	0.031	2.313	0.021^d^
		MS	Genotype	8.096	15.97	2	0.017	0.125	0.046	2.453	0.015^d^
			Allele	6.332	70.22	1	0.012	0.091	0.035	2.523	0.012^d^
		RIS	Genotype	5.672	18.67	2	0.059	0.067	0.046	1.340	0.181^d^
			Allele	1.880	82.18	1	0.170	0.049	0.035	1.371	0.171^d^

a the minimum expected count;

b not assuming the null hypothesis;

c using the asymptotic standard error assuming the null hypothesis;

c based on normal approximation;

IDD: inflammatory demyelinating diseases; MS: multiple sclerosis; RIS: radiologically isolated syndrome.

**Figure 1:**
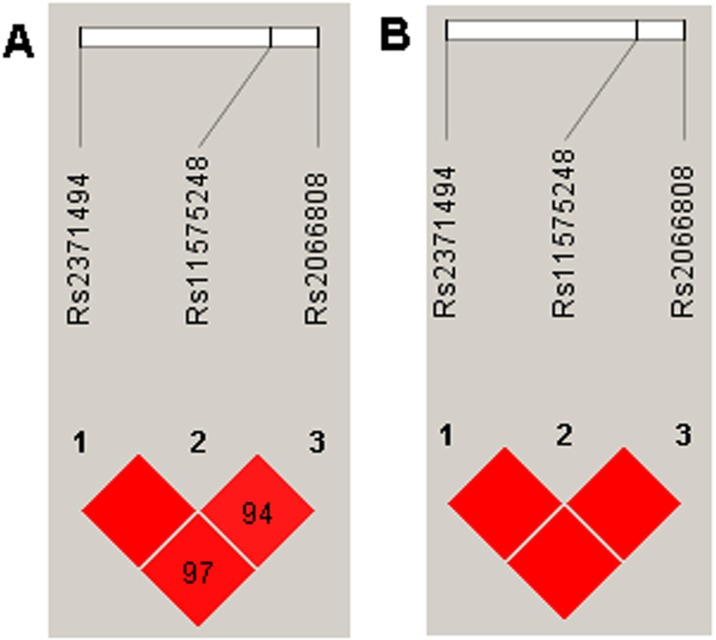
LD analysis of the variants in the IL23A gene region, and the LD plots were generated using the Haploview software v4.2 (**A**) Data analysis between IDD patients and controls from the present study. (**B**) Data from the HapMap CHB. The data from the HapMap CHB and this work were very similar.

The genotype frequencies in the IDD diseases and control groups were further analyzed by three genetic models: additive, dominant, and recessive. The variants rs2066808, rs2371494, rs11575248 and rs1884444 were associated with the risk of IDD diseases, however rs2066808 and rs2371494 were not in the recessive model and rs1884444 was not in the dominant model (Table 4). The experiment-wide significance threshold of the variants rs2066808, rs2371494, rs11575248 is 0.019. The Haploview software was used to conduct LD analysis in the *IL23A* variants, and the results from the LD analysis of the variants (rs2066808, rs2371494 and rs11575248) in the present study and the data from the HapMap CHB population were shown in Fig. 1. The data from the HapMap CHB and this work were consistent. We conducted the Hardy-Weinberg equilibrium test for the IDD diseases and controls and it was in line with equilibrium.

**Table 4:** Analysis of rs2066808, rs2371494, rs11575248 and rs1884444 variants in the IDD and control groups based on three genetic models

Genes	Variants	Additive model (P value)	Dominant model (P value)	Recessive model (P value)
***IL23A***	Rs2066808	0.01731	0.004455	0.5163
	Rs2371494	0.01761	0.008755	1
	Rs11575248	0.01537	0.02242	0.0197
***IL23R***	Rs1884444	0.02291	0.5067	0.001176

IDD: inflammatory demyelinating diseases

### Gene expression analysis

**Figure 2:**
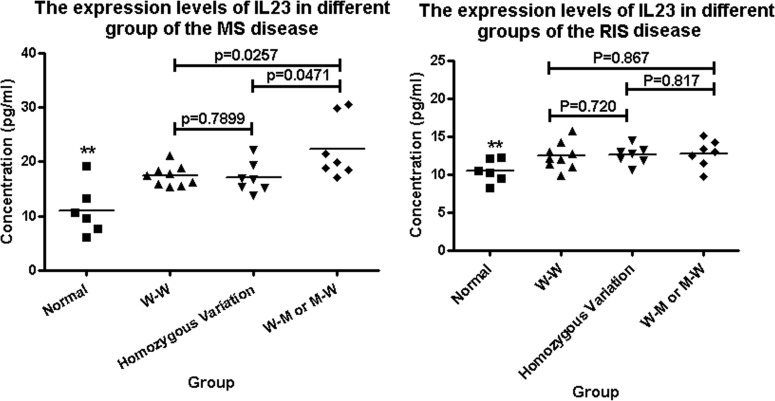
The serum levels of IL23A in IDD and normal groups were detected by ELISA (**A**) The expression levels of IL23A in the MS patients were higher than that in the control group (two asterisk); and when both the *IL-23A* and *IL-23R* genes were altered, the serum levels of IL23A were higher than those in the groups in which neither the *IL-23A* nor the *IL-23R* gene was altered or only one of them was altered. (**B**) The expression levels of IL23A in the RIS patients were higher than those in the control group (two asterisks); however there was no difference between the three groups that had the IL-23A and IL-23R genes both altered, only one altered or neither altered. “WW” means neither the *IL-23A* nor the *IL-23R* gene was altered; “MM” means the *IL-23A* and *IL-23R* genes were both altered; “Homozygous variant” means the homozygous variant G/G of the rs1884444 variant in the *IL-23R* gene.

We conducted the ELISA analysis to measure the serum level of IL23A in the IDD participants and controls between the wild type, heterozygous mutation and homozygous mutation type groups. Firstly, we drew a standard curve and calculated the regression equation (Y = 0.0385X + 0.0393, R2=0.9969; Fig. S2). We found that the serum levels of IL23A in the MS patients were higher than those in the normal group (statistically significant, two asterisks). Notably, when both the *IL-23A* and *IL-23R* genes were altered, the serum levels of IL23A were significantly higher than those in the groups in which the *IL-23A* and *IL-23R* genes were not altered or only one of them was altered (Fig. 2). Remarkably, although the serum levels of IL23A in the RIS patients were higher than those in the normal group (statistically significant), there was no statistically significant difference among the three groups that had the IL-23A and IL-23R genes both altered, only one altered or neither altered (Fig. 2).

## DISCUSSION

In this study, we investigated possible associations of variants in the *IL-23A* and *IL-23R* genes with the risk of inflammatory demyelinating diseases (IDD). We found that three variants (rs2066808, rs2371494, rs11575248) in the *IL-23A* gene and one variant (rs1884444) in the *IL-23R* gene were associated with the risk of IDD, especially MS; the serum levels of IL23A in the MS patients were altered by the co-effects of the variants.

IDDs, especially MS, are deemed to be a kind of autoimmune diseases that develop in individuals with genetic predisposition [15]. The cytokine IL23A has important effects in the differentiation of pro- inflammatory cells, and these cells have been considered to be the main factors in the MS pathogenesis [16]. It has also been reported that more than 90% of MS patients have humoral responses [17], and the serum level of *IL-23A* may reflect autoimmune disease progression and types [18]. Additionally, oligoclonal IgG bands in the cerebrospinal fluid of MS patients are strong predictors for disease course progression [19], and oligoclonal IgM bands in the cerebrospinal fluid of MS patients also are correlated with worse prognosis of the disease [20]. The increased humoral responses also are correlated with MS progression [21], and the subunits of neuro-filament protein may be potential serum and cerebrospinal fluid biomarkers for MS disease progression [22]. In the present work, we found that the serum levels of IL-23A in the MS patients were higher than those in the normal groups. More interestingly, in the group in which both the *IL-23A* and *IL-23R* genes were altered, the serum levels of IL23A were higher than those in the groups in which neither the *IL-23A* nor *IL-23R* genes was altered or only one of them was altered.

Although we found that the serum levels of *IL-23A* in the MS patients were affected by the variants in *IL-23A* and *IL-23R*, these changes were not found in the RIS patients, strongly suggesting differences between MS and RIS in pathogenesis in addition to their differences in clinical characteristics such as onset age, MRI and cerebrospinal fluid (CSF) features, clinical course and morbidity [1] [23] [24]. The reasons underlying those differences between MS and other demyelinating syndromes may be in the balance status of effectors and regulatory immune cells, immune activation, and age-related immune cell access into the central nervous system [25].

As functional genetic variants within the *IL-23A* gene have significant impacts on the host immune response, they could be excellent candidate targets for genetic association studies [26]. The variant rs11171806 located within exon3 of the *IL-23A* gene is significantly associated with the susceptibility to the autoimmune disorder Graves' disease [18]. Although the genetic heterozygosity of the rs11171806 characterized in this study was too low to be useful for susceptibility analysis to MS or other IDD disease, three variants (rs2066808, rs2371494, rs11575248) within the 5-‘UTR or near the 3-‘UTR of the *IL-23A* gene were significantly associated with the risk of MS or other IDD, providing opportunities for etiological and pathogenesis studies.

In addition to the *IL-23A* gene, some genes related to the IL-23A functions also have been investigated for their possible associations with the risk of autoimmune diseases [27, 28], such as the variant rs10889677 in the IL-23R gene associated with MS[29] (consensus not reached yet [30]). In this work, we did not find statistical significance between rs10889677 and MS or other IDD, but another variant rs1884444 within exon1 of the *IL-23R* gene was associated with the risk of MS or other IDD. Of great importance, the variant rs1884444 was associated with changes of the serum levels of IL-23A in the MS patients.

In conclusion, we found that three variants (rs2066808, rs2371494, rs11575248) in the *IL-23A* gene and one variant (rs1884444) in the *IL-23R* gene were associated with the risk of MS and other IDD, and the serum levels of IL-23A in the MS patients were also altered by the variants, providing new evidence for the importance of IL-23A and IL-23R in the pathogenesis of the MS disease and demonstrating the differences between MS and other demyelinating syndromes in pathogenesis.

## MATERIALS AND METHODS

### Study population

We included 206 IDD patients and 300 normal controls (Table 1), which were assembled at the Department of Neurology and Medical Examination Center of the Second Affiliated Hospital of Harbin Medical University, Harbin, China. In order to make the present work consistent with the 1975 Declaration of Helsinki, we obtained a written informed consent from each participant or their guardian, and this work also was approved by the Ethics Committee of Harbin Medical University. We also confirm that all experiments were performed in accordance with relevant guidelines and regulations[31]. Medical histories of the enrolled participants were recorded in detail, and the participants also received physical and neurological system examination. When necessary, some patients also received imageological examination or cerebrospinal fluid inspection. All the patients were diagnosed according to the McDonald's (2010) diagnostic criteria[32].

### DNA analysis

Genomic DNA in peripheral blood leukocytes of each participant was extracted using standard protocols[33]. The transcribed regions and splicing sites of the *IL-23A*, *IL-23R* and *IL-12RB1* genes were amplified by PCR with the primers shown in Table S1. The PCR products were sequenced for mutational analysis using standard protocols [34].

### SNP genotyping analysis and statistical analysis

**Figure 3:**
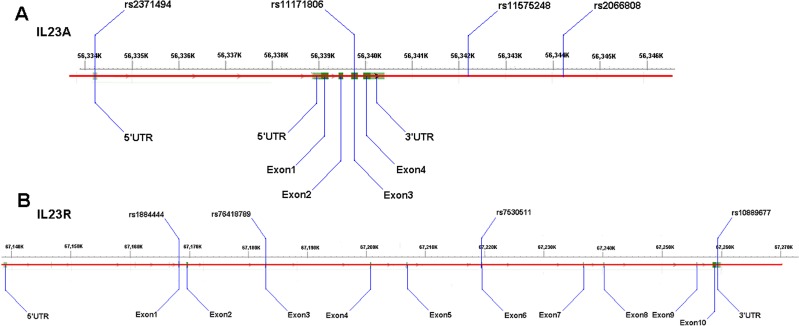
Schematic diagrams of the variants (**A**) the IL-23A gene variants rs2066808, rs2371494 and rs11575248; (**B**): the IL-23R gene variant rs1884444.

The variants within or near the genes of *IL-23A* (rs1884444, rs7530511, rs10889677 and rs76418789), *IL-23R* (rs2066808, rs2371494, rs11575248, rs11171806 and rs401502), and *IL-12RB1* (rs11575934) were determined (Fig. 3) on 206 patients with IDD diseases and 300 normal controls. The DNA regions were amplified and the PCR products sequenced to determine the genotypes. And then overall IDD analysis was conducted according to the types of IDD and sample sizes.

Using the SPSS software (version 19.0), we conducted the statistical analyses [35]. The continuous variables (measurement data, such as age) statistical analyses were conducted using independent-samples T test and the discrete variables (enumeration data, such as gender composition and genotype frequency) statistical analyses were conducted using Chi-Square Tests to calculate P value. P values smaller than 0.05 were considered statistically significant [36]. The Hardy-Weinberg equilibrium tests of the IDD and control populations were conducted with the online software OEGE [37]. The Fisher test was used to compare the genotype frequencies between the IDD and control groups based on three genetic models using PLINK v1.07 software (http://pngu.mgh.harvard.edu/Bpurcell/plink/). We also determined experiment-wide significance threshold, matrix of mpirwise linkage-disequilibrium (LD) correlation for the markers and haplotype diagram of LD structure using SNPSpD software (http://neurogenetics.qimrberghofer.edu.au/SNPSpD/) [38] and Haploview software (http://www.broadinstitute.org/scientific-community/science/programs/medical-and-population-genetics/haploview/haploview) [39].

### ELISA analysis and data calculation

We separated the serum and blood cells and stored the samples at−80°C prior to use. For ELISA assays, we used the Interleukins 23A (Human) ELISA testing kit (96T), No.: CK - E10077H, from Shanghai Yuanye Bio-Technology Co., Ltd. The IL23A levels were measured by ELISA using a microplate reader (BioTeK Epoch, USA).

To make a standard curve for measuring the levels of IL–23A, we put the OD values of six different concentrations of standard substance minus the OD value of blank hole on ordinate Y and the concentrations of the standard substance on the abscissa X, and then calculated the regression equation (Y = aX + b, R2 value). The concentrations of IL–23A in the samples were then determined according to their OD values. Finally, using GraphPad Prism, we conducted the T test.

## SUPPLEMENTARY MATERIAL FIGURES AND TABLE


