# Supplementary material for: Characterization of variations in IL23A and IL23R genes: possible roles in multiple sclerosis and other neuroinflammatory demyelinating diseases
Source: Aging (Albany NY). 2016 Nov 26;8(11):2734–44. doi: 10.18632/aging.101058 (PMC5191866; doi:10.18632/aging.101058)
Supplement: Supplementary file 1 [file aging-08-2734-s001.pdf]

SUPPLEMENTARY MATERIAL

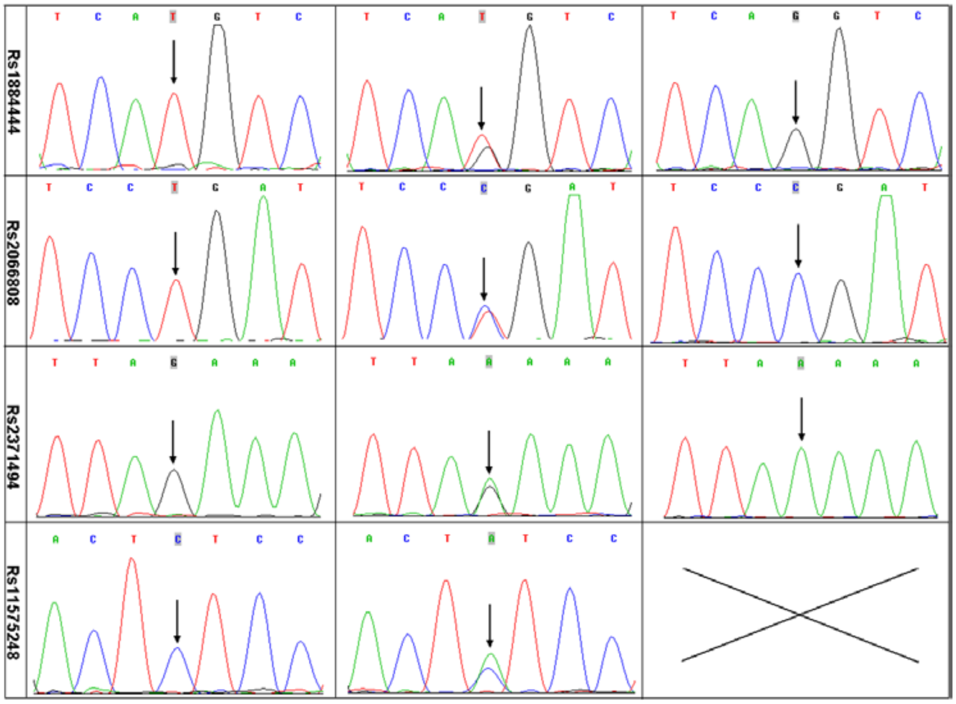

Figure-S1. Genotypes of DNA sequence chromatograms of rs2066808, rs2371494, rs11575248 and rs1884444.

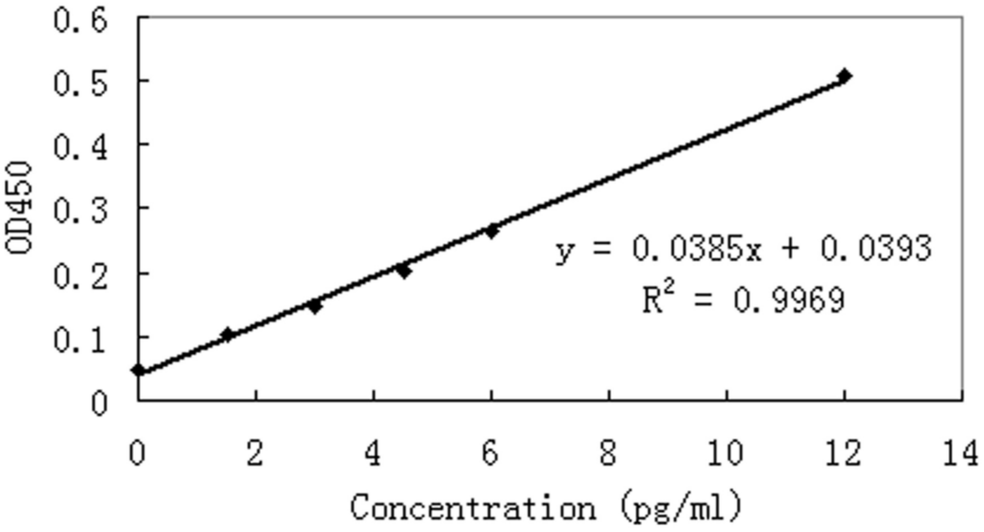

Figure-S2: Standard curve and regression equation (Y = 0.0385X + 0.0393, R^2=0.9969).

Table S1. PCR primers used for *IL23A*, *IL23R* and *IL12RB1* genes sequence analysis.

Gene	*Exon*	*Forward primer*	*Reverse primer*	*Size(bp)*	*Tm (℃)*
IL23A	1	TGAGGGAACCAAACCAGA	CCCTAATGGCCTTCAACC	393	55.4
	2	AGGCTGTGGCAGAAGACC	AAGCAGAACTAGGGAGAAA	448	51.5
	3	CTGAGAACATGGCTGGGTA	CAGGCTGAAAGGACATAGGA	383	53.3
	4	CTGGGCCTCAGCCAACTCCT	CATAGCCCTTCAGAATAGA	700	51.1
IL23R	1	ATCAAAGGTTCCCATCAA	TGTGAGTAAGAGGGCTGT	433	45.8
	2	TGTAATAGGCTAGTTTATG	CAACCTGGCCCTGGAGAC	621	44.9
	3	TGGGAGCTGTAGACTGGA	GAGGCTGATTTCTTGGAT	317	50.8
	4	CAGGCACATGCCACCAAT	GGCAGCAGCACTACTCAT	544	50.2
	5	GATGCCAGTTTCTCCCTA	CACTGATGGTTGGCTTTT	275	48.3
	6	TGATTATGTACGGCCACG	AGCTGCGACTACAAATGC	543	49.5
	7	GATTATAGTTGCCCTTGC	ATCCACCTAAGCCACTTC	511	48.2
	8	GTGTCAGACAAGCCAAAT	GCCTCTTTAAGCCTCATT	692	48.0
	9	AGACAGGGTCTTGCTATG	ATTAGACTGCACTCTTGG	364	47.3
	10	AAATGGAGGGAGAAAGGA	TTCATCAGGAAGCAGGGT	654	49.3
	11	TCAGGAAATAATCCCAGGTT	CCAAGGGAAATGAAAACA	487	48.6
IL12RB1	1	ACCTCGCAGGTGGCAGAG	CCATTTGACAGCAGGAAAGAC	250	57.3
	2	GCATAGATGGGAAAGTGGG	TTTGGCCTGGTGGTGGAG	283	55.6
	3	GGGTTTAGGCTGAGGTGA	AGCAAGAGTGGGATGGTG	278	54.6
	4	GCCTGGGTGACAGAATGA	AATCTAAGTGCCCACGAAT	518	54.7
	5	CTGGACAGGGTGGATTTT	AGATGGTGAGACTGATTTGG	297	54.8
	6	CCAGCCTAGATGACAGAG	GGGTAAGAGGCATACAAA	264	47.4
	7	CAAGGTCACGACCGAAGG	GCTGGGATTACAGGCATCA	579	56.9
	8	GGCAGCAAGGGATTTTCA	GCCCACTTCCTGCCACCT	320	55.2
	9	CTGTCTGCCTATGGGATG	GTGCCTGGCTGATTACTG	460	54.3
	10	CCACCCTGGTTGATAGAG	CAGGTGGATCACTTGAGG	312	50.5
